# Understanding multidisciplinary care for people with rheumatic disease in British Columbia, Canada, through patients, nurses and physicians voices: a qualitative policy evaluation

**DOI:** 10.1186/s12913-021-07138-0

**Published:** 2021-10-23

**Authors:** Glory Apantaku, Magda Aguiar, K. Julia Kaal, Sarah Munro, Michelle Teo, Mark Harrison

**Affiliations:** 1 Faculty of Pharmaceutical Sciences, University of British Columbia, 2405-4625 Wesbrook Mall, Vancouver, BC V6T 1Z3 Canada; 2 Centre for Health Evaluation and Outcome Sciences, St. Paul’s Hospital, Vancouver, BC Canada; 3 Department of Obstetrics and Gynaecology, University of British Columbia, Vancouver, BC Canada; 4 Balfour Medical Clinic, Penticton, BC Canada; 5 Penticton Regional Hospital, Penticton, BC Canada; 6 Clinicial Instructor, Faculty of Medicine, Department of Medicine, University of British Columbia, Vancouver, BC Canada; 7 Arthritis Research Canada, Vancouver, BC Canada

**Keywords:** Fee for service, Canada, Policy, Qualitative evaluation, Rheumatology

## Abstract

**Background:**

In 2011, the province of British Columbia (BC) moved to allow patients with complex rheumatic disease to be seen by nurses along with their rheumatologist by introducing a ‘Multidisciplinary Care Assessments’ (MCA) billing code (G31060).

**Objective:**

To describe multidisciplinary care introduced as part of MCAs across BC and investigate the perceived impact of this intervention, the addition of nurses to the care team, on patient care from the perspective of patients, nurses, and rheumatologists.

**Methods:**

We conducted semi-structured interviews, informed by a qualitative evaluation approach with patients, nurses, and rheumatologists from September 2019 – August 2020. Interviews investigated 1) the experiences of all stakeholders with adopting the multidisciplinary care billing code, 2) the perceived role of the nurse in the care team, and 3) the perceived impact of multidisciplinary care on patient experience and outcomes. We purposefully sampled practices for maximum variation of geographical location (rural vs. urban), size of practice (i.e., patient caseload), and number of nurses employed.

**Results:**

We interviewed 21 patients, 13 nurses, and 12 rheumatologists from across BC. Our analysis identified variation in the way rheumatologists adopted multidisciplinary care across BC. Our analysis showed some heterogeneity in the way the MCA was delivered in rheumatology practices; however, patient education was identified as the core role of nurses across practices. We identified six core themes describing the impact of this model of care, all representing improvements in the way practices functioned, from improved efficiency to access, patient experience, time management, clinician experience, and patient health outcomes. Contextual factors that influenced the presence of these themes were related to the time the nurses spent with patients and the professional roles they performed.

**Conclusion:**

Our results suggest nurse care can complement physician care by extending contact time for patients and promoting the efficient use of health care professionals’ skills, time, and resources. These data may encourage future uptake of the billing code to help ensure the policy delivers maximum benefits to patients given the wide range of perceived benefits described by clinicians and patients.

**Supplementary Information:**

The online version contains supplementary material available at 10.1186/s12913-021-07138-0.

## Introduction

In April 2011, the British Columbia (BC) Ministry of Health introduced a ‘Multidisciplinary Care Assessment’ (MCA) billing code for the care of people with complex rheumatic diseases. The code reimburses a rheumatologist for a greater amount when a nurse is present to provide patient care during the appointment, subject to eligibility criteria. The additional reimbursement allows the rheumatologist to pay for the nurse. Studies exploring inter-professional collaboration in chronic disease care have shown that they result in improved patient outcomes [1, 2] and can improve patient satisfaction with care [2]. Similarly, evidence primarily from randomized controlled trials (RCTs) suggests that multidisciplinary rheumatology care teams can improve the quality and experience of care and lead to better results for patients and the health care system [3]. However, multidisciplinary care involving nurses for patients with rheumatic diseases using the MCA billing code in BC can only be billed for at minimum intervals of 6 months for individual patients. This level of contact hours may be lower than in the examples of successful multidisciplinary care interventions from RCTs [4–7]. Furthermore, while the billing code provides recommendations on best management for people with complex rheumatic diseases, it does not specify what a MCA should include other than recommendations on best management.

An evaluation of the introduction of multidisciplinary care for rheumatology patients through this policy has implications for BC as well as for other Canadian provinces and international jurisdictions looking to introduce similar policies in rheumatic or other chronic diseases. The introduction of the billing code is an example of the type of intervention recommended by the Health Care Innovation Group of Canada’s Premiers [8] and BC Ministry of Health [9] constituting a team-based, inter-professional model of joined-up collaborative care for chronic disease, aimed at improving overall value. In particular, this is a unique billing code in Canada because it allowed rheumatologists working in private and community practices to provide nursing support; other provinces primarily provide nursing support to rheumatologists working in academic rheumatology clinics.

There is limited evidence to inform use of this intervention in BC, and other jurisdictions. The first study to examine the scope of the nurse role in MCAs in BC, a chart review of three urban rheumatology clinics over a period of 3 months in 2012, suggested that nurses were providing education and counselling (about disease and medications) and administering subcutaneous biologics, immunizations, and tuberculosis skin tests [10]. Further research in 2018, again in three urban rheumatology clinics, indicated that the introduction of nursing support under the MCA billing code increased the number of weekly outpatient encounters per rheumatologist by 75%, suggesting that nurses were playing a role in increasing accessibility of care for patients [11]. By 2018, over 70% of the 77 rheumatologists in BC were billing for the MCA and use of the billing code increased by over 100% from 2015 to 2020, suggesting that multidisciplinary care in rheumatology services is widespread [11, 12]. However, to date there has been no comprehensive study of the use of this multidisciplinary care billing code describing how nurses have been integrated into rheumatology clinics, their role in the care team, or the perceived impact of multidisciplinary care from the perspective of all stakeholders.

Our study uses a qualitative evaluation approach to investigate how rheumatologists have incorporated multidisciplinary care into their practice and the perceived impact of this intervention on patient care from the perspective of patients, nurses and rheumatologists. We chose a qualitative evaluation approach due to its strength in providing nuanced understanding of how individual and interpersonal behavior contributes to successful implementation of evidence-based interventions [13].

## Methods

### The intervention

An MCA billing code (G31060) which paid a premium to reimburse rheumatologists for providing multidisciplinary care with a nurse for patients with complex rheumatic was introduced in British Columbia (BC) during the financial year 2011/12 as part of the Specialist Services Committee (SSC) fees [14]. Full details of the MCA code are provided in Supplement 1.

### Approach

We employed a qualitative evaluation approach due to its ability to provide important contextual data to ensure a well-rounded understanding of the contribution of individual and interpersonal behavior to successful implementation of evidence-based interventions in different environments and policy contexts [13]. We conducted the project in collaboration with patient groups (Arthritis Consumer Experts/Joint Health; Arthritis Research Canada Arthritis Patient Advisory Board), rheumatologists (BC Society of Rheumatologists), rheumatology nurse organizations (BC Rheumatology Nurses Society), and the BC Ministry of Health.

### Setting and recruitment

Rheumatology practices that employ a nurse under the MCA billing code were eligible for inclusion. A self-administered, online survey conducted by the BC Society of Rheumatologists (BCSR) of all Royal College of Physicians and Surgeons of Canada certified rheumatologists in BC included a question asking whether respondents would be willing to be contacted to participate in this study and to provide a contact email. We subsequently sent email invitations via the BCSR to the 27 (53%) rheumatologists who reported employing a nurse and had expressed interest in participating. We invited them to contact us if they were willing to be interviewed for this study. We purposefully sampled practices for maximum variation in geographical and urban/rural location (at least one practice from each health authority in BC), size of practice, number of nurses employed, and length of time nurses have been employed.

At each participating practice we asked the rheumatologist or their medical office assistant (MOA) to invite the nurse to participate. We scheduled interviews with nurses at a time and place that was convenient and suitable to nurses, and could be face-to-face or by telephone. We recruited patients through two methods; (1) the MOA or rheumatologist provided verbal information about the study to patients, (2) the study was advertised to patients by our patient partner groups Arthritis Consumer Experts (ACE) and Arthritis Research Canada’s Arthritis Patient Advisory Board (APAB). ACE is a national patient-led organization based in Vancouver BC providing free information and education to patients through multiple media platforms [15]. APAB is a board of patient volunteers supported by Arthritis Research Canada ( Vancouver, BC) who aim to represent the patient perspective on research and communicate research findings back to patients [16]. If patients expressed an interest by providing their contact details, we sent an introductory letter, and then our research coordinator contacted them, screened them for eligibility, and then scheduled an interview. We interviewed participants either in person or over the phone. The study was reviewed and approved by the UBC Behavioral Research Ethics Board (BREB) (H19–00258), and recruitment of patients adhered to UBC BREB’s Guidance Note 17.1.1 on Identifying and Contacting prospective Subjects from Primary Health Care Provider Records.

### Interview guide

Our interview guide explored the characteristics of multidisciplinary rheumatology care in BC, and the perceived impact on patient care. We developed the interview guide using information from a variety of sources, including responses to the BCSR survey, a literature review of the potential roles of rheumatology nurses and models of care, and guidelines from Europe recommending a role for nurses in rheumatology care [17, 18]. We piloted the interview guide with members of the study team, which include rheumatologists, rheumatology nurses, and patients. We also reviewed the fit, clarity, and comprehensiveness of the guide after the first interviews with each participant group and made minor semantic changes as needed [19].

### Data collection

We interviewed participants in person or over the phone from June 2019 – August 2020. Two female researchers (MA and GA) conducted the interviews. Both interviewers were experienced in in-depth interviewing and qualitative methodology, and sought to be attuned to the participants’ comfort level throughout the interview process. MA is a pharmacist and postdoctoral fellow, and GA is a public health researcher. Neither interviewer had experience living with a rheumatoid disease or caring for people with one, but they sought to develop rapport through honesty, curiosity, and empathy. The interview process was detailed and balanced, we used open ended questions to guide participants to explore both benefits and harms. We explicitly sought to inquire on possible harms by asking “What are the main (if any) disadvantage(s) of seeing a nurse?” We continued data collection until theoretical data saturation was achieved, interviews did not generate new insights regarding the implementation of multidisciplinary care in rheumatology in BC, and there was sufficient data to provide a rich, nuanced understanding of key implementation domains [20]. We audio-recorded interviews and used a professional transcription service to transcribe interviews; minor edits were made to remove potentially identifying information about staff and their clinics.

### Data analysis

Our analysis involved coding interviews inductively using an iterative, thematic approach and guided by the objectives of the study [21]. We conducted our analysis using NVivo 12 software (QSR International) and involved line-by-line coding of transcripts followed by constant comparison to identify patterns. GA and KJK conducted an initial review of transcripts to gain familiarity with the data, identify preliminary themes and patterns, and develop a coding framework. They piloted the initial coding framework by coding two interviews and iteratively reviewing their coding together. Additionally, they each independently coded a sub-set of transcripts to refine the codebook. Disagreements were resolved through discussion with a third researcher (MA). Finally, they synthesized and interpreted themes through discussion with the co-authorship team and wrote the results into an explanatory narrative.

## Results

We conducted 46 interviews with 21 patients, 12 rheumatologists, and 13 nurses from a total of 11 practices. The groups of patients and nurses were primarily female (85%), while we had an equal proportion of males and females amongst our rheumatologists. In all groups we had good representation of participants from urban (minimum 38%) and rural/remote areas (minimum 44%). Most patient participants were aged between 35 and 65 years, but included patients aged 65 and above (24%). The rheumatologists and nurses had between 1 and 9 years of experience working within the MCAs.

In the following sections we provide a narrative summary of the analysis of interviews and supporting quotes.

### Characteristics of multidisciplinary rheumatology care in BC

Overall there was considerable heterogeneity in the way MCAs were arranged, however all models were physician-led. Rheumatologists described learning about the billing code mostly from other rheumatologists who provided the social influence that led them to use the code and adopt multidisciplinary care.

#### Appointment structure

Appointment structure varied, but broadly could be described as one of three structures, examples are shown in Table 1. Firstly, in some practices, appointments were sequential and then shared. The nurses would start the appointment alone with the patient, completing standard assessments (e.g. the Health Assessment Questionnaire) and enquiring about their general wellbeing. The rheumatologist then joined the consultation, at which point it became a three-way shared conversation between the rheumatologist, nurse, and patient. The second common structure saw appointments arranged in a sequential structure with minimal overlap; the nurses gathered information from the patient and relayed it to the rheumatologist, who finished the consult alone. The third structure mirrored the second, but allowed the nurse to finish the appointment with the patient, providing patients a chance to debrief. The appointment structures had varying benefits, from providing nurses the opportunity to clarify and buttress information to patients after their consult with the rheumatologist to ensuring patients had their nurses present during their appointment, increasing the opportunity for adequate follow-up and continuity of care.

*“I'll go in. I'll see the patient, do the update of information. The doctor will then go to see them and decide, ‘Okay. Yes, they do need to start on this medication or start subcutaneous methotrexate.’ Then they’ll come get me and say, ‘I need you to go back into that room and do some teaching.’ Then I'll go back in and do that after they’ve seen the doctor.”* – *Nurse 25*

*“Dr. [redacted] will join the appointment, and I’ll give her a bit of a summary of what the patient and I have discussed. We do that together, in front of the patient, so it’s like a summary and a paraphrase. Then if I misinterpret it, then it gives the patient an opportunity to correct me.” – Nurse 45*

Additionally, the reported duration of appointments varied, with an average follow-up visit during which a patient sees both the nurse and rheumatologist ranging from 15 min to 60 min. Nurses also reported variations in the time they spent alone with patients, ranging from 5 min to 45 min.

**Table 1:** Characteristics of Multidisciplinary Care using three sample practices

	Case A (R6 and N7)	Case B (R10 and N45)	Case C (R40 and N27)
**Appointment Flow**	Sequential visits Nurse only 10 min [Handover] Rheumatologist 10 min [Handover] Nurse only 5 min (debrief)	Shared visits Nurse only 5–10 min Rheumatologist + Nurse 5–15 min Nurse only 5 min	Sequential visits Nurse only 20–30 min [Handover] Rheum only 20–30 min
**Appointment duration**	15–30 min	20–30 min	40–60 min
**Role of nurses** • Patient education • Emotional support	**Rheumatologist:** “There’s a myriad of stuff that the nurses do for us, a lot of education, a lot of counselling, a lot of dealing with comorbidities, a lot of issues around medication adherence.” **Nurse:** “I see my role as an educator to provide the patient with the most up-to-date information, whether it’s about their medication or their disease processes or pharmaceutical, non-pharmaceutical interventions to kind of help them gain the skills that they need to work through this life-long condition”	**Rheumatologist:** “My nurses identify gaps in patient care that need to be dealt with and we look to make sure patients are adherent. The nurses will take about adherence, side effects and a way to mitigate it. There are many, many, many opportunities during every single visit in which the nurse, the nurses provide added care to patients” **Nurse:** “I think of myself as a patient educator is what I think of myself as.”	**Rheumatologist:** “It saves time. I don’t have to go through every single medication with the patient … and then I think the counselling part— “You need to take this vaccine and that vaccine”— so again, that would save time and add to patient care.” **Nurse:** “Education is the biggest one, I think. Then also, sometimes they’ll talk to me more than the doctor. I don’t know why, but they will open up more.”

#### Social and professional role of nurses – patient education

The core role nurses played in this model of care was identified as patient education, which included education on disease and medications, as well as more holistic self-management, lifestyle, and wellbeing strategies. Rheumatoid diseases are complex, with wide ranging effects on the overall wellbeing of patients. The presence of nurses providing education on lifestyle factors like smoking cessation and pregnancy planning was a very welcome addition. Our patient participants appreciated the addition of nurses to the care team as it provided them with an opportunity to access more comprehensive information to support their disease management.. Nurses were described by patients and rheumatologists as being able to “translate” the information that rheumatologists provided into a more digestible form for patients. Nurses identified with this role, seeing it as a core part of their scope of practice. Patients and rheumatologists also appreciated and valued nurses for their attentiveness and their ability to provide opportunities for patients to share information they may have been unable to or uncomfortable sharing with rheumatologists. Additionally, nurses provided emotional support to patients, and assisted the rheumatologist with charting and documentation.

*“I see my role as an educator to provide the patient with the most up-to-date information, whether it’s about their medication or their disease processes or pharmaceutical, non-pharmaceutical interventions to kind of help them gain the skills that they need to work through this life-long condition” – Nurse 7*

*“[S] he always talks to me about non-medicine ways to manage my pain. Exercise and yoga and things like that. You know, counselling and education is what she primarily does.” – Patient 46*

### Impact of multidisciplinary Care on patients, physician, and nurses

We identified six core themes that describe how the Multidisciplinary Care Assessments impacted patient care. Perceptions and experiences were primarily positively, hence these themes illustrate the strengths and value of multidisciplinary care from the perspective of patients, nurses, and rheumatologists.

#### Increased access to care for patients

Patients described having more access to their rheumatology practices. Both patients and rheumatologists experienced the increased access resulting from the presence of a nurse as a stress relieving factor. For patients, they appreciated the assurance of timely response to their questions and concerns. For rheumatologists, their shorter waitlist alleviated their concerns about providing timely care and support to patients.

*“we have access to our nurses, like, by e-mail. If we have a question or something in between time, we can e-mail the question and they’ll answer us back.” – Patient 23*

*“you can call, and the nurse will literally will call you back and answer the question rather than you having to not waste the doctor’s time.” – Patient 41*

*“it improves access because the same one physician can see more patients in terms of number, and more patient visits, so that improves access because other people can get in so it improves our wait lists” - Rheumatologist 34*

#### Efficiency: getting the most out of each appointment

Clinicians and patients noted that the practice functioned more efficiently. The increased efficiency and smooth running of the practice and appointments had direct implications for patient and rheumatologists. Patients experienced their appointment as been more informative and comprehensive, they noted that the presence of the nurse helped them get the most out of their appointment.. Rheumatologists also discussed experiencing more moments of flow, as they were less distracted by administrative and other tasks.

*“I am happy with that because it’s more efficient for him and for everybody.” – Patient 3*

*“with a nurse, I find things just go smoother. Like, I’m talking to my doctor, and then maybe the nurse has done some record keeping and gotten my prescriptions and everything ready. Over there, so it just makes the appointment smoother and faster. And, um, we deal with fatigue, so having shorter appointments, faster appointments, well-organized appointments really helps” – Patient 37*

*“the nurses really helped me be more efficient in seeing my patients. I can do the rheumatology portion of the visit, and the nurse could help with a whole bunch of the rest of the visit.” – Rheumatologist 34*

#### Making more time for patients

Rheumatologists and patients reflected specifically on the effects of the presence of nurses on how their time was spent. The ability to have a more rewarding time in their work for rheumatologists and a more enlightening time during their appointment for rheumatologist was cherished by both stakeholders. Rheumatologists felt they were able to spend more time on the parts of their job they truly enjoyed (i.e., patient care). Patients valued the time spent with nurses as it increased their interaction with a healthcare professional and relived some of the burden on their rheumatologist.

*“What’s nice, too, about with having the nurses, you go in. They ask you how you’re doing. They’re very attentive. They write down the information, the questions that need to be asked. This has saved so much time for rheumatologists to see more patients on their limited time.” – Patient 22*

*“The most valuable thing is that you don’t feel rushed. You have the time to express whatever feelings or concerns, and you don’t feel rushed because you have that time to discuss.” – Patient 42“The nurses free up my time to do other things because they can do the education and teaching on certain drugs … Patients, you know, respond really well to it.” – Rheumatologist 33*

#### Patients experience more holistic care

Overall, patients felt cared for and ‘heard’, they perceived their care as more holistic and reported having a more pleasant experience at their appointments when a nurse was involved. They appreciated the increased interaction with a healthcare professional during their appointment and the feeling of knowing there was always someone ready to support them when they needed it.

*“with many of the members on my care team is that even if I don't have to utilize them the fact that I know that they're there if I need them. It gives us peace of mind, which reduces the stress level and anxiety.” – Patient 30*

*“I think there’s more caring, not just paperwork, she’s brought definitely a more—I don’t know—a more pleasant experience, I would say” - Patient 41*

#### Improved patient health outcomes

Nurses and rheumatologists experienced the multidisciplinary care model as providing improved patient health outcomes. They believed the increased education, support and follow up patients received from nurses, in addition to the opportunity to freely share concerns and have their questions answered resulted in increased adherence to treatment regimen.

*“So, having a nurse got through it, spend a lot of time with them makes them much more adherent to the treatment.” – Rheumatologist 14*

*“I know 100 percent that our immunization rates in our patients is higher now with our nurses because they are always checking it and they are updating it and making sure that they are doing it.” – Rheumatologist 5*

*“There’s many people who don’t take the recommendations initially, and then, after time, do, so it’s just with those repetitive nurse-client interactions that they sort of open up to it.” – Nurse - 11*.

#### A ‘happier’ team experience

Rheumatologists experienced a positive impact on their emotions, describing feeling happier since working with a nurse. They appreciated having someone to share their workload and valued the more intricate understanding that nurses seemed to have of patients’ psychosocial issues. Nurses also appreciated being able to work in rheumatology practices and felt their presence contributed positively to work-life balance for all team members.

*“Then I think the benefit for Dr. [redacted] is that her workload is more manageable. So that she also can have that healthy work-life balance because she has the nursing support in her office.” – Nurse 45*

*“I think it helped my practice, helped the quality of care, helped our satisfaction, so we’re all way happier. Way happier.” – Rheumatologist 16*

We sought to elicit negative impacts of MCAs, however our participants noted that even the “side-effects” turned out to be beneficial. Patients mentioned that although appointments sometimes lasted longer, taking more time from their day, they appreciated it as they got more out of the appointment. Rheumatologists noted that it took time to get their nurses ‘fully up to speed’ but even then, their presence was still a positive addition and once they were fully integrated the benefits of having them increased.

*“A lot of inefficiency and loss of productivity for the first six months, but then we figured it out, and we worked together, but you get what you put into it … You have to be willing to give up that time, energy, and money to invest in your nurse. Then you can really maximize on the services that a nurse can provide, the added value that they can provide to a clinic.” – Rheumatologist 10*

*“I like having a longer appointment. Sometimes it’s harder to get to them because, you know, you’re having to try to find the time between work to get to it. But, I enjoy, once you can find that time, to have the longer appointment. I like them way better.” – P41*

## Discussion

This study sought to qualitatively evaluate the impact of having nurses work with rheumatologists in a multidisciplinary team through the introduction of MCA billing code on rheumatology care in BC. Our study provides a nuanced understanding of how stakeholders have experienced the MCA and the possible pathways by which multidisciplinary care can impact patient outcomes. We observed that rheumatologists saw more patients, possibly due to the fact that practices were reported as running more efficiently. Patients felt they got more out of their appointments and the care they received was more holistic. Rheumatologists and nurses also perceived that their patients had better outcomes. From the patient perspective, our analysis showed that patients place a high value on having more face-time with a health professional, they appreciated the thorough conversations they had with their nurses on medications and overall health along with recommendations on modifying disease management; this additional facetime with a health professional was informative and thus empowering for patients. Overall, our results indicate that the addition of multidisciplinary care through a fee-for-service billing code has changed the care rheumatology patients receive in BC, and has been received well by all stakeholders involved.

Our findings that the addition of nurses to care teams through a fee-for-service billing code can result in nurses providing patient education and counselling and equipping patients to manage their disease better is important for policy-makers. Previous research exploring the needs and experiences of patients in nursing care and the role of nurses in a multidisciplinary care model identified this as the most beneficial role nurses played in the practice given its effect of equipping patients to manage their disease better [22–24]. However, much of these benefits have previously been delivered in tightly controlled clinical settings [22] or reported studies with strict protocols [22, 23]. Guidelines for optimal care of rheumatology patients from the UK and Europe recommend that all patients with inflammatory arthritis should receive education about the medication and self-management; our findings suggest that rheumatologists and nurses are achieving this in BC through the MCA billing code. There are parallels with incentive schemes in primary care in the UK, which led to nurses assuming responsibility for continuity of care for people with chronic diseases [25], and primary care practices achieving better scores on measures of patient outcomes [26].

There is evidence that nurse consults which include dedicated teaching can improve patients’ health behaviors [27], adherence to treatment [28], and understanding of the potential adverse effects of treatment [29]. The MCA provided an opportunity for rheumatologists to be innovative and tailor the mode of care delivery to their patient population. We found that nurses were able to provide a range of educational resources to patients including options for smoking cessation, pregnancy planning, vaccinations, and social support. Additionally,; nurses and rheumatologists observed that patients adhered better to medications as they had increased access to detailed information and an opportunity to discuss concerns with a nurse. Studies of medication adherence in rheumatology have shown that it is a significant concern and a challenging issue to address [28, 30]. However, if the reports of improved adherence in our results are accurate, the MCA could be contributing to patients being better able to manage and control their disease [31]. Our results emphasize the fact that for patients with lifelong conditions like rheumatoid diseases, the education and support they receive from their healthcare professionals has an impact on their ability to manage their disease and its wide-ranging effect on their lives. Our findings suggest that codes similar to the MCA for rheumatology could be introduced to support the care of people with other chronic diseases.

The delivery of multidisciplinary rheumatology care varied across practices and their characteristics were determined by rheumatologists’ preferences on how to organize their practice. Our analysis indicates that in a three-way conversation nurses were able to clarify and simplify information for the patient, which might be a way of attenuating power and agency imbalances in health care. However, it is not known if a model is superior to another, or what additional benefits come from having a level of guidance on implementation from the ministry that allows practices to make adaptations based on their geographic and population characteristics. The lack of direction on use of the MCA code in the guidance from the BC Ministry of Care payment schedule [14] might mean that some rheumatologists maximize the full potential of the code whilst others do not. However, opportunities to share experiences among rheumatologist might help to promote more consistent and effective use of the code. Future research could explore these variations in more depth and provide useful evidence for any future development of guidelines on how to implement multidisciplinary care in BC. Similarly, if maximizing the potential of the MCA code is related to characteristics of rheumatology care that are already associated with higher levels of access to rheumatology services, for example working in larger rheumatology practices, then it is possible that it could contribute to broadening inequalities in care provision in BC. Traveling rheumatologists may have limited opportunities to provide MCA given the logistics of their appointments and thus the patients who see them, who often already struggle with accessing specialist care may be unable to benefit from this model of care. It would be useful to study any possible negative effects of this policy such as widening of inequities with patients in urban areas having the benefits and patients in rural and remote areas unable to receive this model of care.

Rheumatologists in our study valued the MCA for the increased access it provided their patients, and felt their practices were more responsive to patients and their waitlists shorter. This supports previous findings from a study of three rheumatology offices that showed the MCA allowed rheumatologists to see more patients [11]. Rheumatologists also felt, they were able to focus on the most rewarding aspects of their jobs. This had a positive effect on morale with rheumatologists reporting a significant improvement in their emotional experience at work as they were able to share the burden and provide a better experience for their patients.

A strength of this study is the fact that it was conducted by independent researchers. Additionally, our interview process was rigorous, we sought to guide patients to explore both benefits and harms that may have resulted from the multidisciplinary care by explicitly asking “What are the main (if any) disadvantage(s) of seeing a nurse?” The core limitation of this study is potential social desirability bias as patients were recruited from rheumatology practices and may have felt the need to speak positively about the practice. Additionally, our sample possibly includes the most engaged and responsive set of practices as they indicated an interest to participate in a survey. We sought to mitigate social desirability bias by explicitly stating that responses would be completely confidential and used only for research purposes, not for quality improvement and feedback for the practices, as well as having arms-length trainees from the research team conduct data collection. A further limitation was the fact that we were unable to recruit patients from remote areas in BC and therefore did not capture what could very well be a geographically specific experience unique to patients in these areas. However, all clinicians we interviewed, including those serving patients in rural and remote areas, reported similar experiences with the MCA billing code.

We also did not interview any clinicians who had not adopted the MCA. Our recruitment mechanism asked rheumatologists who reported having a nurse if they would be willing to participate. For this reason, we are unable to provide the perspectives of rheumatologists who did not use the MCA billing code on their reasons for not using the code or any limiting factors that prevented their use of the MCA.. It is also possible that the few practices who have not adopted the MCA in any form may have a different patient population with less complex patients, and are therefore not eligible for the billing code [11]. Nevertheless, collecting data to help understand reasons why the MCA code is not always adopted would be valuable to provide details on this subset of practices.

In conclusion, this study sheds light on the implementation of multidisciplinary care programs in specialist services. Our results demonstrate that nurse care can complement physician care and add value to patient and clinician experience by improving access for patients, promoting efficient use of professionals’ skills, and improving patient health outcomes. Furthermore, reporting on MCA implementation and the benefits of employing a nurse may encourage future uptake in provinces across Canada and an expansion of the billing code to other chronic disease.

## Supplementary Information

**Additional file 1.**

## Data Availability

The datasets generated during and/or analyzed during the current study are available from the corresponding author on reasonable request and subject to ethics approval.

## Abbreviations

BC: British Columbia
BCSR: British Columbia Society of Rheumatologists
BREB: Behavioral Research Ethics Board
LPN: Licensed Practical Nurse
MCA: Multidisciplinary Care Assessment
MOA: Medial office assistant
MoH: Ministry of Health
MSP: Medical Services Plan
RCT: Randomized Controlled Trial
RN: Registered Nurse
SSC: Specialist Services Committee
