# Supplementary material for: Understanding multidisciplinary care for people with rheumatic disease in British Columbia, Canada, through patients, nurses and physicians voices: a qualitative policy evaluation
Source: BMC Health Serv Res. 2021 Oct 23;21:1148. doi: 10.1186/s12913-021-07138-0 (PMC8542329; doi:10.1186/s12913-021-07138-0)
Supplement: Supplementary file 1 — Additional file 1. [file 12913_2021_7138_MOESM1_ESM.docx]

| **Specialist Services Committee Initiated Listings (Section of Rheumatology**[14]**)**  **G31060 - Multidisciplinary Conference for community-based patients ($225.96)** |
| --- |
| To consist of assessment, written treatment plan and any other counselling the patient needs for management of their particular diagnosis. |
| Notes:   1. Restricted to rheumatology 2. For the ongoing management of complex disorders of the musculoskeletal system, where the complexity of the condition requires the continuing management by a rheumatologist. It is not intended for the evaluation and/or management of uncomplicated rheumatologic disorders (e.g. osteoarthritis, bursitis/tendonitis, neck and back pain). 3. Only paid when a Registered Nurse or Licensed Practical Nurse is present. 4. Applicable to patients with rheumatoid arthritis diagnoses or similar inflammatory disease 5. Maximum one per patient in 6-month period. 6. Not paid in addition to 31010 (Consultation: $212.47), 31012 (Repeated or Limited Consultation: $120.96), 31007 (Subsequent office visit: $$88.74) or G31050 (Extended consultation-exceeding 53 minutes: $270.47) |
